# Pathogenic Lineage of *mcr*-Negative Colistin-Resistant *Escherichia coli*, Japan, 2008–2015

**DOI:** 10.3201/eid2212.161117

**Published:** 2016-12

**Authors:** Toyotaka Sato, Akira Fukuda, Yuuki Suzuki, Tsukasa Shiraishi, Hiroyuki Honda, Masaaki Shinagawa, Soh Yamamoto, Noriko Ogasawara, Masaru Usui, Hiroki Takahashi, Satoshi Takahashi, Yutaka Tamura, Shin-ichi Yokota

**Affiliations:** Sapporo Medical University School of Medicine, Sapporo, Japan (T. Sato, Y. Suzuki, T. Shiraishi, H. Honda, S. Yamamoto, N. Ogasawara, H. Takahashi, S. Takahashi, S. Yokota);; Rakuno Gakuen University, Ebetsu, Japan (A. Fukuda, M. Usui, Y. Tamura);; Sapporo Medical University Hospital, Sapporo (M. Shinagawa)

**Keywords:** Escherichia coli, bacteria, pathogen, colistin, Japan, multidrug resistance, antimicrobial resistance

**To the Editor:** Colistin is a last-line drug for treatment of multidrug-resistant, gram-negative bacterial infections, including those caused by *Escherichia coli*. We report colistin-resistant *E. coli* isolates from Japan, including a global-spreading pathogenic lineage, serotype O25b:H4, sequence type (ST) 131, and subclone *H*30-R (O25b:H4-ST131-*H*30R).

We tested 514 *E. coli* isolates obtained from clinical specimens taken at Sapporo Clinical Laboratory Inc. (Sapporo, Japan) and Sapporo Medical University Hospital in Japan during 2008–2009 (*1*) and 2015, respectively. Samples were processed according to Clinical and Laboratory Standards Institute guidelines (*2*). Identification of O25b:H4-ST131, O25b, H4, and ST131 were determined as described previously (*1*). For identification of the *H*30Rx subclone of O25b:H4-ST131, *H*30 was determined by PCR using a specific primer set (*3*), R was determined according to ciprofloxacin MIC, and x was determined by detecting 2 single-nucleotide polymorphisms, as previously described (*4*).

Four *E. coli* isolates exceeded the colistin resistance breakpoint (>2 mg/mL) (Table). None of the patients from whom the *E. coli* isolates were derived had a history of colistin treatment. Three of the 4 colistin-resistant isolates belonged to a pandemic lineage, O25b:H4-ST131-*H*30R, which has been isolated from urinary tract and bloodstream infections (*3*,*4*). The frequency of colistin-resistant ST131 *E. coli* isolates among O25b:H4-ST131 was 2.2%. This lineage is fluoroquinolone resistant and is frequently resistant to β-lactams because it possesses CTX-M–type extended-spectrum β-lactamase genes (*1*,*3*,*4*). 

**Table:** Characterization of colistin-resistant *Escherichia coli* isolates, Japan, 2008–2105*

Strain	Specimen type	Patient age, y/sex	Year	Serotype	ST	MIC, mg/L†									
						PIP	CAZ	CPD	FEP	IPM	GEN	AMK	CIP	CST	PMB
SRE34	Urine catheter	UNK/F	2008	O25b:H4	131- H30Rx	128 (R)	1 (S)	1 (S)	0.06 (S)	0.125 (S)	0.5 (S)	2 (S)	32 (R)	16 (R), 16 (R)‡	8, 8‡
SRE44	Urine catheter	UNK/M	2008	O25b:H4	131- H30Rx	64 (R)	2 (S)	1 (S)	0.13 (S)	0.25 (S)	0.5 (S)	1 (S)	64 (R)	16 (R), 16 (R)‡	16, 8‡
SME222	Indwelling pericardial drain	76/M	2015	O18	416	2 (S)	0.5 (S)	0.5 (S)	<0.03 (S)	0.13 (S)	0.5 (S)	2 (S)	0.03 (S)	4 (R), 4 (R)‡	8, 4‡
SME296	Urine	67/M	2015	O25b:H4	131- H30R	>128 (R)	32 (R)	>128 (R)	16 (R)	0.13 (S)	0.5 (S)	2 (S)	64 (R)	4 (R), 8 (R)‡	1, 4‡

*All isolates were phylogentic group B2. AMK, amikacin; CAZ, ceftazidime; CIP, ciprofloxacin; CPD, cefpodoxime; FEP, cefepime; CST, colistin; GEN, gentamicin; IPM, imipenem; PIP, piperacillin; PMB, polymyxin B; R, resistant; S, susceptible; ST, sequence type; UNK, unknown. †EUCAST (http://www.eucast.org/) breakpoints were used for resistance determination because the colistin breakpoint for *E. coli* was undetermined by the Clinical and Laboratory Standards Institute. MICs were determined by the agar dilution method unless otherwise stated. Breakpoints: PIP, >16; CAZ, >16; CPD, >1; FEP, >4; IPM, >8; GEN, >4; AMK, >16; CIP, >1; CST, >2. ‡Broth microdilution method.

The colistin-resistant isolates reported were resistant to fluoroquinolones, and 1 (SME296) was resistant to cephalosporins (due to expression of *bla*_CTX-M-14_). Another colistin-resistant *E. coli* isolate (SME222) belonged to O18-ST416, which is also known as an extraintestinal pathogenic *E. coli* (*5*), although this lineage has not previously been reported to exhibit colistin resistance.

The colistin-resistant *E. coli* isolates we identified were sensitive to carbapenems and aminoglycosides, including amikacin, whereas previously it was reported that some *E. coli* ST131 isolates exhibited resistance to carbapenems by possessing carbapenemases, such as NDM-1 and KPC-2; the NDM-1–possessing ST131 isolate also exhibited resistance to amikacin (*6*,*7*). Thus, these findings may affect future antimicrobial choices because of the clonal dominance, multidrug resistance, and pathogenicity of the isolates.

Recent studies reported a plasmid-mediated colistin resistance gene, *mcr-1*, in various countries (*8*). In addition, a novel plasmid-mediated colistin resistance gene, *mcr-2* (76.7% nucleotide identity to *mcr-1*), was found in *E. coli* isolates in Belgium (*9*). These genes encode a phosphoethanolamine transferase family protein, which modifies the lipid A component of lipopolysaccharide (*8*,*9*). The colistin-resistant *E. coli* isolates we identified did not possess *mcr-1*or *mcr-2*, although the MICs for colistin were the same as or higher than that of the transconjugant of a *mcr-1*–harboring plasmid in an *E. coli* ST131 isolate (4 mg/L) reported by Liu et al. (*8*). Thus, these colistin-resistant isolates may have other colistin resistance mechanisms. For example, modification of lipid A with 4-amino-4-deoxy-l-arabinose or phosphoethanolamine, caused by chromosomal mutations in *mgrB*, *phoPQ*, and *pmrAB* genes, might occur and could be responsible for the resistance. This polymyxin-resistance mechanism is seen in *Enterobacteriaceae*; however, other novel mechanisms are also conceivable.

In conclusion, we report colistin resistance in a major global-spreading extraintestinal pathogenic *E. coli* strain, O25b:H4-ST131-*H*30R, in Japan. This strain acquired colistin resistance without carrying a plasmid bearing the *mcr* gene. Clarifying the colistin-resistance mechanisms in these isolates is necessary if we are to forestall the emergence of multidrug (including colistin)–resistant O25b:H4-ST131-*H*30R. The worst-case scenario is the global spread of this isolate, which has acquired resistance to the last-line antimicrobial drug, colistin.

## References

[1] Yokota S, Sato T, Okubo T, Ohkoshi Y, Okabayashi T, Kuwahara O, et al. Prevalence of fluoroquinolone-resistant *Escherichia coli* O25:H4-ST131 (CTX-M-15-nonproducing) strains isolated in Japan. Chemotherapy. 2012;58:52–9. 10.1159/000336129 22343392

[2] Clinical and Laboratory Standards Institute. Performance standards for antimicrobial susceptibility testing (M100-S25). Wayne (PA): The Institute; 2015.

[3] Colpan A, Johnston B, Porter S, Clabots C, Anway R, Thao L, et al.; VICTORY (Veterans Influence of Clonal Types on Resistance: Year 2011) Investigators. *Escherichia coli* sequence type 131 (ST131) subclone *H*30 as an emergent multidrug-resistant pathogen among US veterans. Clin Infect Dis. 2013;57:1256–65. 10.1093/cid/cit503 23926176 PMC3792724

[4] Price LB, Johnson JR, Aziz M, Clabots C, Johnston B, Tchesnokova V, et al. The epidemic of extended-spectrum-β-lactamase-producing *Escherichia coli* ST131 is driven by a single highly pathogenic subclone, *H*30-Rx. MBio. 2013;4:e00377–13. 10.1128/mBio.00377-13 24345742 PMC3870262

[5] Lau SH, Reddy S, Cheesbrough J, Bolton FJ, Willshaw G, Cheasty T, et al. Major uropathogenic *Escherichia coli* strain isolated in the northwest of England identified by multilocus sequence typing. J Clin Microbiol. 2008;46:1076–80. 10.1128/JCM.02065-07 18199778 PMC2268376

[6] Peirano G, Schreckenberger PC, Pitout JD. Characteristics of NDM-1-producing *Escherichia coli* isolates that belong to the successful and virulent clone ST131. Antimicrob Agents Chemother. 2011;55:2986–8. 10.1128/AAC.01763-10 21444703 PMC3101395

[7] Morris D, Boyle F, Ludden C, Condon I, Hale J, O’Connell N, et al. Production of KPC-2 carbapenemase by an *Escherichia coli* clinical isolate belonging to the international ST131 clone. Antimicrob Agents Chemother. 2011;55:4935–6. 10.1128/AAC.05127-11 21768521 PMC3186997

[8] Liu YY, Wang Y, Walsh TR, Yi LX, Zhang R, Spencer J, et al. Emergence of plasmid-mediated colistin resistance mechanism MCR-1 in animals and human beings in China: a microbiological and molecular biological study. Lancet Infect Dis. 2016;16:161–8. 10.1016/S1473-3099(15)00424-7 26603172

[9] Xavier BB, Lammens C, Ruhal R, Kumar-Singh S, Butaye P, Goossens H, et al. Identification of a novel plasmid-mediated colistin-resistance gene, mcr-2, in Escherichia coli, Belgium, 2016. Euro Surveill. 2016;21. 10.2807/1560-7917.ES.2016.21.2730280 27416987

